# Impact of enhanced recovery after surgery (ERAS) protocols on clinical outcomes of patients undergoing anterior cervical discectomy and fusion: A retrospective study

**DOI:** 10.1097/MD.0000000000049523

**Published:** 2026-07-03

**Authors:** YanMing Hou, Dingli Xu, Leling Feng

**Affiliations:** a Department of Orthopedics, Deqing People’s Hospital, Huzhou, Zhejiang, China; b Department of Clinical Medicine, Health Science Center, Ningbo University, Ningbo, Zhejiang, China; c Department of Nursing, Ningbo No.6 Hospital, Ningbo, Zhejiang, China.

**Keywords:** anterior cervical discectomy and fusion, clinical effect, enhanced recovery after surgery, opioid usage, surgery

## Abstract

This study aimed to evaluate the impact of enhanced recovery after surgery (ERAS) protocols on the clinical outcomes of anterior cervical discectomy and fusion (ACDF). This retrospective study included eligible patients with 3-level cervical disc herniation treated with ACDF between April 2020 and April 2022, with a minimum 1-year follow-up. Patients were categorized into 2 groups based on whether they received an ERAS protocol (ERAS+ group) or standard perioperative care (ERAS− group). Key outcome measures included hospital length of stay, total cost, estimated blood loss, visual analogue scale pain scores, Neck Disability Index, Japanese Orthopaedic Association scores, perioperative opioid usage, complication rates, and Bridwell bone fusion grades. Statistical analyses were performed using Statistical Package for Social Sciences 24.0, with significance set at *P* < .05. The implementation of an ERAS protocol in patients undergoing multilevel ACDF may be associated with improved perioperative metrics, enhanced functional recovery, reduced opioid use, and lower complication rates in this retrospective analysis. Further prospective studies are warranted to confirm these observations. A total of 110 eligible patients were enrolled in our study. Compared with the ERAS− group, the ERAS+ group demonstrated significantly shorter hospital stays (3.6 ± 0.5 vs 4.3 ± 0.8 days, *P* < .05), lower total costs (18,569.2 ± 872.4 vs 21,201.9 ± 1160.8 CNY, *P* < .05), and reduced estimated blood loss (75.5 ± 14.6 vs 107.2 ± 27.5 mL, *P* < .05). The ERAS+ group also showed superior improvements in visual analogue scale, Neck Disability Index, and Japanese Orthopaedic Association scores at follow-up visits (*P* < .05). Furthermore, the ERAS+ group had significantly lower perioperative opioid consumption, a lower incidence of overall complications (*P* < .05), and better Bridwell fusion grades (*P* < .05).

## 1. Introduction

Anterior cervical discectomy and fusion (ACDF) has been regarded as the safest and most commonly performed cervical surgery for patients with cervical spine disc herniation. However, ACDF often results in postoperative pain, heightened perioperative opioid usage, and delayed mobilization of cervical.^[1,2]^ Dysphagia, odynophagia, and even dyspnea were also reported in some published studies.^[3,4]^ Nowadays, researchers reckon that enhanced recovery after surgery (ERAS) protocols could be a possible method that may decrease complications, cost, and hospital stay after operation.^[5,6]^

ERAS protocols are a combination of perioperative approaches to optimizing care with a multidisciplinary evidence-based framework that accelerates recovery, minimizing physiological stress and hastening functional restoration after major surgery, with the added benefit of reducing total costs.^[7–9]^ The idea of ERAS was widely applied to the laparotomy procedure in general surgery. Zhang et al reported that they compared the clinical outcomes of 114 patients who underwent major surgery, 57 patients intervened with ERAS were the intervention group and 57 patients without ERAS were the regular group, and found that the there was a reduction in the incidence rate of postoperative complications, hospital stay, and total cost in the intervention group compared with the regular group (*P* < .05).^[10]^ With the promotion of the concept of ERAS, some researchers propose that ERAS may have a positive effect on patients who underwent lumbar surgery. Porche et al reported that among 114 patients who were treated with open transforaminal lumbar interbody fusion (TLIF), 57 implemented with ERAS (ERAS group) and the other 57 patients without ERAS (pre-ERAS group), patients in the ERAS group were associated with decreased operative time, reduced length of stay, decreased opioid consumption, and improved physiological outcomes for open TLIF (*P* < .05).^[11]^ Similarly, Band et al reported that they compared 33 patients treated with minimally invasive lumbar fusion surgery (16 were in ERAS group and 17 were in control group), and found that ERAS application to minimally invasive TLIF was safe and effective, significantly reducing long of hospital stay and inpatient opioid consumption(*P* < .05).^[12]^

ERAS has the potential to improve patient outcomes and satisfaction from a variety of different angles; however, existing studies focus on lumbar operation, and only few studies about the effect of ERAS protocol on patients who underwent ACDF. So, in this study, we hypothesized that patients treated with ACDF with ERAS protocol (preoperative specialist consultations, psychological assessments, perioperative opioid-sparing strategies, and other components) may experiencereduced complications, along with improved Japanese Orthopaedic Association (JOA) and Neck Disability Index (NDI), compared with patients without ERAS.

## 2. Methods

This retrospective analysis was conducted using data from a prospectively collected, single-center database that included patients treated with ACDF from April 2020 to April 2024 at our Hospital. Before enrollment, Institutional Review Board approval was obtained (2025-36L), and all participants provided informed consent. The study population consisted of adult patients aged 18 years or older who underwent surgical treatment for more than a 3-level cervical spine disc herniation.

All cases were diagnosed based on symptoms, physical examination, and radiology (magnetic resonance imaging), and failure to achieve minimal 3 months of conservative treatment. Our inclusion criteria were as follows: age >18 years old, 3-segment cervical spine disc herniation treated with ACDF, minimal 1-year follow-up visit with complete data (complete data at 1-year follow-up was defined as the availability of all prespecified outcome measures: visual analogue scale (VAS), NDI, JOA scores, radiographic assessment of fusion [Bridwell grade], and record of any complications), and with typical symptoms such as neck or upper limb pain with numbness of fingers. The exclusion criteria were as follows: history of cervical surgery; spinal-related diseases such as fracture, scoliosis, spinal tumor, and cervical spine deformity; and rheumatic arthritis, cervical tuberculosis, and osteoporosis or other systemic diseases such as chronic renal failure that may influence preoperative recovery.

The present study followed the Declaration of Helsinki. Informed consent was obtained from all participants, and their information would be stored and used for research anonymously. This study was approved by the Institutional Review Boards and Ethics Committee of Deqing People’s Hospital. The patients provided written informed consent to participate in this study.

## 3. ERAS protocols

ERAS protocols consisted of a patient undergoing all or some of the following: patient education on enhanced recovery strategy, preoperative medication reconciliation, analgesia optimization, physical therapy, methods of venous thromboembolism prophylaxis, preoperative tracheal traction, perioperative antimicrobial prophylaxis, adequate fluid management and nutrition, early Foley catheter removal, enhanced discharge planning including faster time-to-ambulation goals, and early follow-up after discharge. The details of the ERAS protocol are shown in Table 1.

**Table 1:** ERAS protocol.

Protocol	
Preoperative	Nutritional optimization (diabetes, dietary management)
	Specialist consult: dysphasia risk, cardiology evaluation
	Chronic medication reconciliation
Perioperation	Osteoporosis management: patients with osteoporosis or at high risk of osteoporosis, based on a DEXA scan with a *T*-score <−1.1, will be offered oral drugs, administered for 3 months. Patients with age of >80 were excluded
	Opioid-sparing strategies
	Venous thromboembolism prophylaxis
	Early mobilization with physical therapists: patients started to stand, walk with a cervical collar with rehabilitation physiotherapist’s help
	Intensive care unit avoidance or minimization
	Enhanced wound care protocol and expedited catheter/drain removal when possible: prophylactic antibiotics (cefazolin sodium) are administered at 2 mg/d every 8 h. If the drainage is <30 mL/d, the drains are removed
Postdischarge	Expedited discharge
	Care team virtual follow-up (discharge day + 1)
	Pain-management consultation as soon as possible post-op
	Pain-management consultation as soon as possible postoperative and in-office consultation (2–3 wk postdischarge)

DEXA = dual energy X-ray absorptiometry, ERAS = enhanced recovery after surgery.

### 3.1. Group allocation and ERAS protocol

After our department implemented ERAS, the patients who were willing to participate in the study received ERAS. Before implementing this policy, conventional methods were used.

### 3.2. Surgery procedure

All patients were anesthetized with intratracheal intubation in the supine position and treated with 3-segment ACDF by one medical group. The incision was a 2- or 3-finger breadth above the clavicle and along the medial border of the left sternocleidomastoid. When the longissimus colli muscle and pretracheal fascia were exposed, the pathological segment was located by C-arm. After the intervertebral disc and cartilaginous endplate were completely removed, autologous corticocancellous bone was used for grafting. Finally, intervertebral cages (VENTURE, Medtronic, Minnesota), plates, and screws (VENTURE) were inserted for fixation and fusion. After the internal fixation was confirmed in a satisfactory position under C-arm, soft tissues were closed layer by layer, and a drain was placed.

### 3.3. Rehabilitation principle

All patients wore neck collars to maintain cervical stability, and clinical and radiographic assessments were taken at each follow-up visit. Neurotrophic drugs and painkillers were used according to the patients’ symptoms. Patients were supported to avoid long-standing or physical work. Finally, patients were allowed back to the outpatient department to record their clinical outcomes (including VAS, JOA, and NDI) and remove the cervical collar within their toleration at a 3-month follow-up visit, as well as at a 1-year follow-up visit.

### 3.4. Outcome evaluation

Baseline characteristics, including age, gender, cost, smoking, and diabetes, were collected from medical records, and operation-related parameters (hospital stay, blood loss, and complications) and clinical outcomes (NDI, JOA, and C2 to C7 range of motion [ROM]) were also collected.

Radiological outcomes were measured by 2 experienced radiologists, and the details are as follows: the ROM, which is the Cobb angle between the C2 and C7 vertebrae in the extension and flexion positions. Moreover, bone fusion grades were analyzed based on the Bridwell fusion grading system^[13]^ (Table 2).

**Table 2:** Bridwell interbody fusion grading system.

Grade	Description
I	Fused with remodeling and trabeculae present
II	Graft intact, not fully remodeled and incorporated, but no lucency present
III	Graft intact, potential lucency present at the top and bottom of graft
IV	Fusion absent with collapse/resorption of graft

As for clinical outcomes, neck pain was assessed using the NDI,^[14]^ JOA scores were used to evaluate the daily living abilities of patients.^[15]^ VAS was regarded as a direct parameter for a patient’s pain, which was obtained at preoperative, immediately after operation, and 3-month follow-up visit. What’s more, C-reactive protein (CRP) was also analyzed in this study.

Complications were collected according to medical records. Complications that adversely affected the patient’s recovery or required intervention were defined as major complications such as suppuration of incision, leakage of cerebrospinal fluid, intraspinal hematoma, internal fixation failure, bone delay union, venous thromboembolism, and wound and pulmonary infection. The others were minor complications, including redness and swelling of the incision and pain in the incision.

In order to better judge the clinical significance of the difference between the 2 groups, minimally clinically important differences (MCID) were also used. According to already-published articles, the MCID of JOA was set as 2,^[16]^ and the MCID of the NDI was set as 7.5.^[17]^

## 4. Statistical analysis

Statistical Package for Social Sciences 24.0 (IBM Corp., Armonk) was used for the statistical analysis. All data were expressed as mean ± standard deviation. The Shapiro–Wilk test was used to determine the normality of continuous data. Age, ROM, NDI, and JOA measurement data were compared using analysis of variance. The categorical data were compared using the χ^2^ test. *P* < .05 was considered statistically significant. A Kaplan–Meier survival curve was used to illustrate the relationship of the pain-kill drug usage with time in both groups. A log-rank *P* value <.05 was considered statistically significant.

## 5. Results

### 5.1. Baseline characteristics

**Figure 1.**
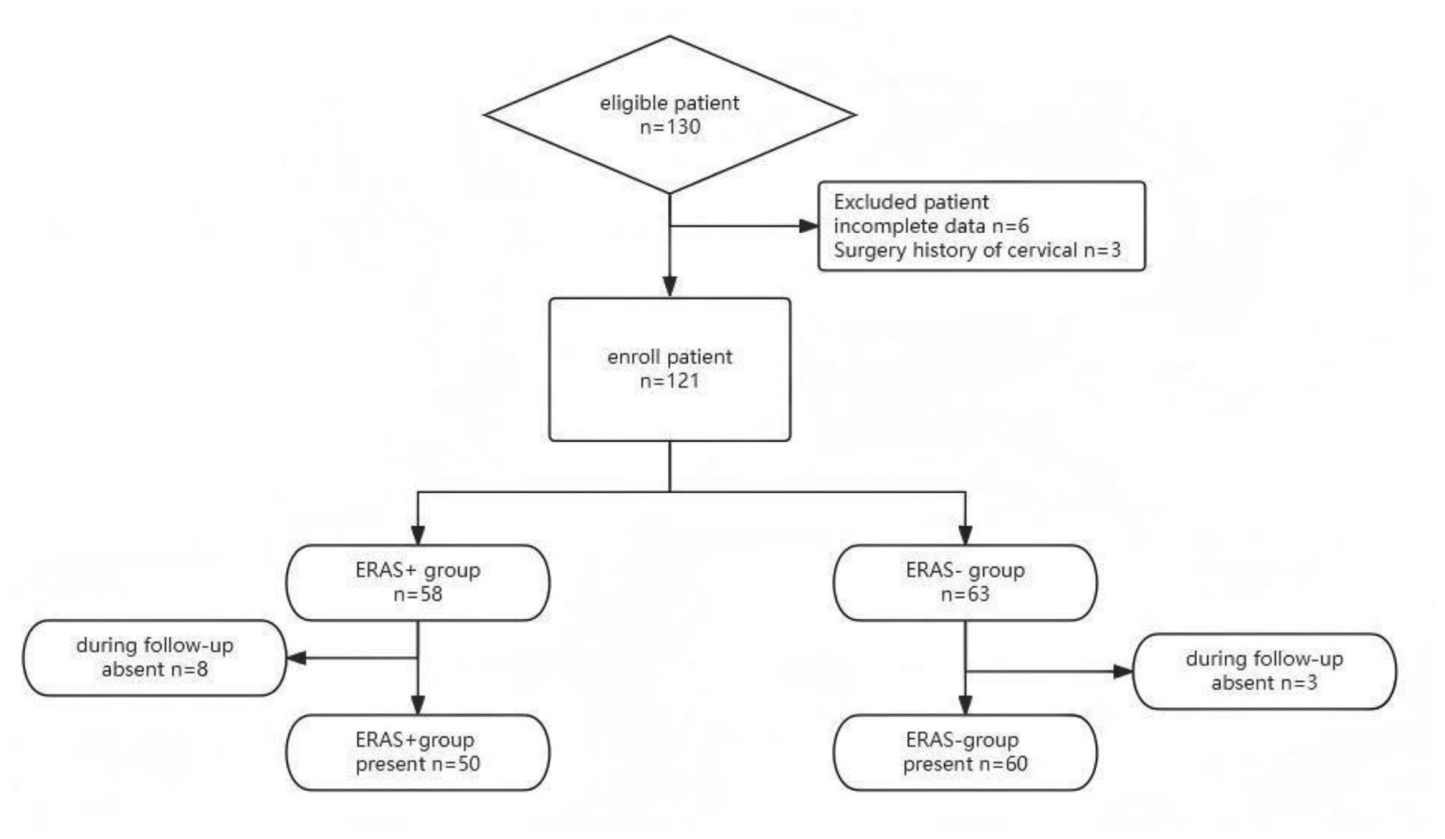
The flow chart of the study. ERAS = enhanced recovery after surgery.

We enrolled 110 ACDF patients who met our criteria in this study (Fig. 1) and retrospectively reviewed their medical records. Significantly shorter hospital stay (3.6 ± 0.5 days vs 4.3 ± 0.8 days; *P* < .05), less blood loss (75.5 ± 14.6 mL vs 107.2 ± 27.5 mL; *P* < .05), and lower cost (18,569.2 ± 872.4 CNY vs 21,201.9 ± 1160.8 CNY; *P* < .05) were observed in the ERAS+ group than in the ERAS− group. There were no significant differences in baseline demographics, including age, gender distribution, smoking status, or prevalence of diabetes between the 2 groups (all *P* > .05, Table 3).

**Table 3:** The comparison of baseline characteristics between 2 groups.

	ERAS+	ERAS−	*P* value
Number	50	60	
Gender			
Male	22	26	.55
Female	28	34	
Age (yr)	66.0 ± 7.6	67.4 ± 6.4	.87
Smoke			.51
Yes	13	10	
No	47	45	
Diabetes	12	8	.39
Yes	48	42	
No			
Hospital stay (d)	3.6 ± 0.5	4.3 ± 0.8	<.05
Cost	18,569.2 ± 872.4	21,201.9 ± 1160.8	<.05
Blood loss (mL)	75.5 ± 14.6	107.2 ± 27.5	<.05
Blood pressure	145.6 ± 15.7	165.8 ± 24.8	<.05
Intra-operation propofol use	232.5 ± 24.9	286.5 ± 33.5	<.05

ERAS = enhanced recovery after surgery.

### 5.2. Clinical outcomes

**Figure 2.**
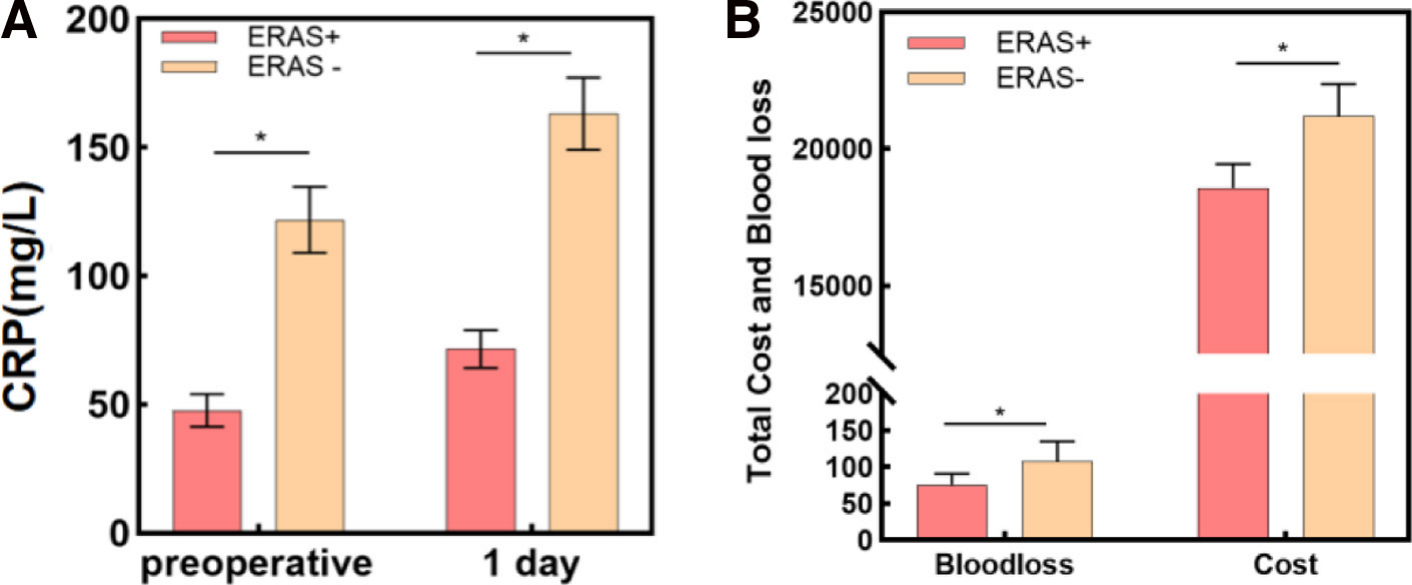
(A) The comparison of CRP between 2 groups at preoperative and 1 day after operation. (B) The comparison of cost and blood loss between 2 groups. CRP = C-reactive protein.

**Figure 3.**
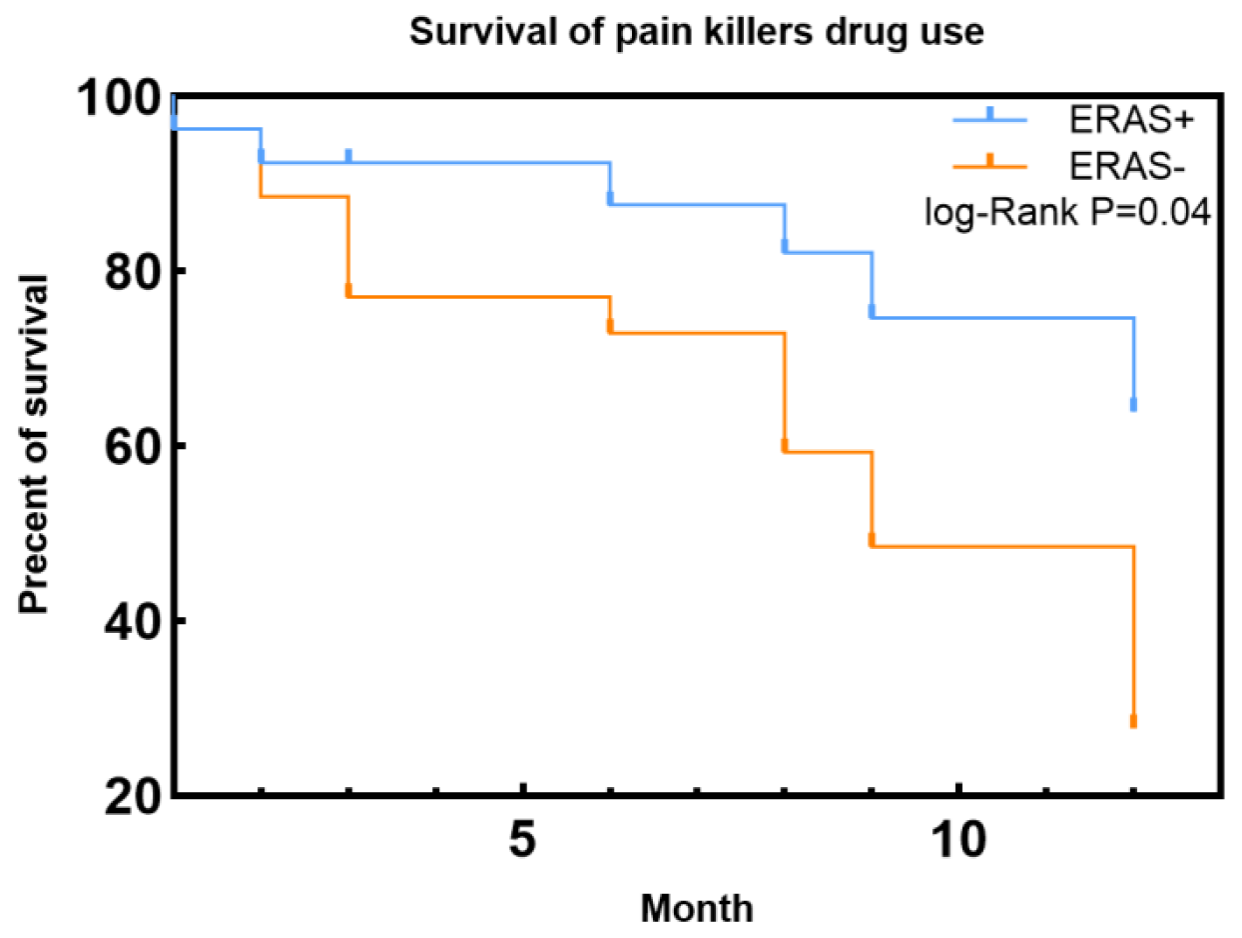
The KM curve of the pain killer drug use in 2 groups. ERAS = enhanced recovery after surgery, KM = Kaplan–Meier.

The clinical outcomes are shown in Table 4. There were no significant differences in VAS, JOA, and NDI between the 2 groups preoperatively (*P* > .05). During the follow-up visit, the ERAS+ group achieved significantly better VAS, NDI, and JOA scores than the ERAS− group (*P* < .05, Table 4). As shown in Figure 2, the ERAS+ group showed significantly lower CRP at preoperative (47.7 ± 6.3 mg/L vs 121.9 ± 12.8 mg/L; *P* < .05) and 1 day after operation (71.6 ± 7.3 mg/L vs 163.1 ± 14.1 mg/L; *P* < .05, Fig. 2) than the ERAS− group. As for ROM, the ERAS+ group showed significantly better results than the ERAS− group during the follow-up visit time (*P* < .05). As for the usage of pain-kill drugs, the Kaplan–Meier survival curve was used (Fig. 3), and the results showed that the usage of pain-kill drugs in the ERAS− group was significantly more than in the ERAS+ group (odds ratio = 2.99, 95% confidence interval: 1.194–7.46; *P* < .05).

**Table 4:** The comparison of clinical outcomes between 2 groups.

	ERAS+	ERAS−	*P* value
Number	50	60	
JOA			
Preoperation	10.0 ± 1.4	9.7 ± 1.2	.27
1-month follow-up	12.7 ± 0.9	10.3 ± 1.3	<.05
6-month follow-up	13.6 ± 1.3	12.3 ± 1.1	<.05
1-year follow-up	16.0 ± 0.8	14.2 ± 1.0	<.05
NDI			
Preoperation	50.2 ± 5.9	50.3 ± 6.0	.93
1-month follow-up	41.9 ± 6.3	46.8 ± 6.5	<.05
6-month follow-up	38.2 ± 5.6	40.7 ± 5.6	<.05
1-year follow-up	21.6 ± 2.0	29.5 ± 4.4	<.05
ROM			
Preoperation	23.6 ± 3.8	23.6 ± 3.6	.58
1-month follow-up	19.0 ± 3.8	20.0 ± 3.4	<.05
1-year follow-up	15.5 ± 3.3	17.5 ± 3.3	<.05
VAS			
Preoperation	6.1 ± 0.9	6.0 ± 0.8	.62
Immediately after operation	4.6 ± 1.1	5.6 ± 1.2	<.05
3-month follow-up	1.1 ± 0.5	2.2 ± 0.89	<.05

ERAS = enhanced recovery after surgery, JOA = Japanese Orthopaedic Association scores, NDI = Neck Disability Index, ROM = C2 to C7 range of motion, VAS = visual analogue scale.

### 5.3. Complication and bone fusion grade

As for the complication and bone fusion grade (Table 5), a total of 15 complications were observed in the ERAS− group and 4 patients in the ERAS+ group, which showed a significant difference between the 2 groups (*P* < .05). Precisely, in the ERAS− group, 5 patients suffered from incision exudation and 4 had incision redness and swelling, all of whom were successfully managed by conservative treatment and antibiotic prophylaxis. One patient showed internal fixation loosening, which was successfully managed by wearing a cervical collar for a longer period; 2 had venous thromboembolism; and 3 had pulmonary infection, who underwent cefoperazone sodium and sulbactam sodium 4.5 g/8 h based on the recommendations from the clinical pharmacist. In the ERAS+ group, 2 patients suffered from incision exudation, and 1 had incision redness and swelling, all of whom were successfully managed by conservative treatment, and 1 had pulmonary infection, who underwent cefoperazone sodium and sulbactam sodium 4.5 g/8 h based on the recommendations from the clinical pharmacist. What’s more, ERAS+ group achieved significantly better in the Bridwell Bone fusion grade than the ERAS− group (*P* < .05, Table 5).

**Table 5:** The comparison of complications and Bridwell fusion grade between 2 groups.

	ERAS+	ERAS−	*P* value
Complication			<.05
Incision exudation	2	5	
Incision red and swollen	1	4	
Fixation loosen	0	1	
Venous thromboembolism	0	2	
Wound and pulmonary infection	1	3	
Bridwell bone fusion grade			<.05
I	46	40	
II	3	18	
III	1	2	
IV	0	0	

ERAS = enhanced recovery after surgery.

## 6. Discussion

This retrospective study aimed to evaluate the impact of an ERAS protocol on outcomes in patients undergoing multilevel ACDF. Our key findings indicate that the ERAS+ protocol was associated with several significant benefits, including reduced hospital stay, lower costs, decreased blood loss, improved short- and medium-term pain and functional scores (VAS, NDI, JOA), lower postoperative inflammatory marker (CRP) levels, reduced opioid consumption, fewer complications, and better radiographic fusion grades.

ERAS protocols are a set of interventions that are carried out in the perioperative period to improve clinical outcomes of patients and accelerate recovery after surgery.^[18]^ The concept of ERAS was first applied to colorectal surgery; however, ERAS protocols have been used in spine surgery to decrease surgical complications and minimize opioid use.^[19]^Yao et al reported a prospective study about 242 patients who underwent lumbar spinal fusion and found that the ERAS group had significantly shorter operative time (202 ± 68 minutes vs 255 ± 85 minutes) and estimate blood loss (480 ± 302 mL vs 641 ± 387 mL) compared with the non-ERAS group (*P* < .05). Moreover, the ERAS group had significantly less total morphine-sulfate-equivalent consumption (27 ± 24 mg vs 42 ± 42 mg; *P* < .05) than the non-ERAS group.^[20]^ In the same way, Han et al reported that for 333 elder patients undergoing 1- or 2-level lumbar fusion surgery, the estimated blood loss of the ERAS-elder group (n = 113) was significantly less than the no-ERAS-elder group (n = 120; 187.2 ± 160.39 mL vs 250.5 ± 195.44 mL; *P* < .05), and the ERAS group exhibited earlier first ambulation (1.78 ± 1.39 days vs 2.48 ± 1.57 days; *P* < .05).^[21]^ Nowadays, ACDF has been regarded as a safe and effective procedure for treating cervical radiculopathy or myelopathy. But problems such as postoperative pain, heightened perioperative opioid usage, and even dyspnea were also reported.^[22,23]^ And ERAS could be one of the possible methods to solve the above problems.

In this study, we compared the clinical outcomes of 50 patients (ERAS+ group) and 60 patients (ERAS− group), and there were no significant differences in age, gender, smoking, and diabetes between the 2 groups (*P* > .05). During follow-up visits, significantly shorter hospital stays (3.6 ± 0.5 days vs 4.3 ± 0.8 days; *P* < .05), less blood loss (75.5 ± 14.6 mL vs 107.2 ± 27.5 mL; *P* < .05), and lower cost (18,569.2 ± 872.4 CNY vs 21,201.9 ± 1160.8 CNY; *P* < .05) were observed in the ERAS+ group than in the ERAS− group. Similarly, outcomes were reported by Wang et al, who reported that for 161 patients who underwent lumbar tubular microdiscectomy (85 in the pre-ERAS group and 76 in the post-ERAS group), the hospitalization costs (16,089.7 ± 3892.7 vs 17,359.9 ± 5762.4; *P* < .05) and length of stay (5.1 ± 1.2 vs 6.2 ± 1.6; *P* < .05) in the post-ERAS group were significantly lower than in the pre-ERAS group.^[24]^

As for clinical outcomes, we found that there were no significant differences in VAS, JOA, and NDI between the 2 groups preoperatively (*P* > .05). During follow-up visits, the ERAS+ group achieved significantly better VAS, NDI, and JOA scores than the ERAS− group (*P* < .05). Similarly, Passias et al reported that for 131 patients who underwent adult cervical deformity surgery, the ERAS group achieved significantly improved modified JOA (Japanese Orthopedic Association scores) at 6 weeks (*P* < .05),^[25]^ Moreover, Tretiakov et al reported that 127 patients underwent cervical deformity surgery, and found ERAS+ group achieved significantly lower in total cost (35,008.38 ± 4012.39 vs 49,031.13 ± 28039.33; *P* < .05) than the ERAS− group (*P* < .05).^[26]^

The level of CRP was also analyzed in our study, as shown in Figure 1. The ERAS+ group achieved significantly lower in CRP level than the ERAS− group, which suggested a reduction in inflammation, indicative of less pain, tissue damage, and less depression after surgery.^[27]^ The same outcome was also reported. Mogalli et al reported that 185 patients underwent total knee arthroplasty (128 in the ERAS+ group, 57 in the control group). The ERAS+ group showed significantly lower CRP levels (55.88 ± 42.60 mg/L vs 94.89 ± 64.17 mg/L; *P* < .05) than the control group at 3 days after operation.^[28]^

ERAS can decrease the usage of pain-kill drugs during the perioperative period. We compared the usage of propofol intra-operation and found that significantly less propofol use (232.5 ± 24.9 mL vs 286.5 ± 33.5 mL; *P* < .05) was observed in the ERAS+ group than in the ERAS− group. Similarly, significantly lower contract blood pressure (145.6 ± 15.7 mm Hg vs 165.8 ± 24.8 mm Hg; *P* < .05) at preoperation was observed in the ERAS+ group than in the ERAS− group. Tretiakov et al also reported that among 220 patients who underwent adult cervical deformity corrective surgery (54 in the ERAS+ group, 166 in the ERAS− group), significantly more usage of propofol was found in the ERAS− group (*P* < .05).^[29]^ The possible reason for less propofol usage is that the ERAS protocol can significantly decrease state-anxiety scores, so the preoperative blood pressure was also lower than in patients without ERAS,^[30]^ and more propofol needed to be used for managing the higher blood pressure in the ERAS− group.^[31]^ What’s more, we also analyzed the usage of painkiller drugs during the follow-up visit period. As shown in Figure 2, the Kaplan–Meier curve indicates that the risk of using opioid in ERAS− group was 1.36 times than ERAS+ group (*P* < .05). Jogerst et al reported that 756 patients who underwent mastectomy were evaluated (405 ERAS− and 351 ERAS+), and found there were decreased odds of chronic opioid use (odds ratio = 0.57, 95% confidence interval: 0.42–0.76) for patients in ERAS+ group.^[32]^

### 6.1. Limitations

This study has several limitations. First, its retrospective, single-center design is inherently susceptible to selection and information biases, despite our attempts to control for them through strict inclusion/exclusion criteria. The non-randomized allocation of patients to ERAS or standard care, based on the timing of protocol implementation, introduces the possibility of residual confounding. Second, the absence of a multivariable analysis to adjust for potential confounding factors limits the strength of causal inference. Third, the study focused exclusively on 3-level ACDF, limiting the generalizability of findings to other cervical procedures or fewer surgical levels. Therefore, the observed associations should be interpreted as preliminary, and definitive conclusions regarding causality require validation through prospective, randomized controlled trials.

## 7. Conclusion

In conclusion, this retrospective analysis suggests that the implementation of a comprehensive ERAS protocol for patients undergoing multilevel ACDF may be associated with substantive benefits, including improved perioperative efficiency (shorter stay, lower cost), enhanced functional recovery, diminished opioid usage, and lower complication rates. These findings support the potential value of ERAS in cervical spine surgery and highlight the need for further rigorous prospective studies to confirm its efficacy and elucidate optimal protocol components.

## Author contributions

**Data curation:** Dingli Xu.

**Writing – original draft:** YanMing Hou.

**Writing – review & editing:** Leling Feng.
